# A consideration of the social dimensions and implications of neuroimaging research in global health, as related to the theory-ladened and theory-generating aspects of technology

**DOI:** 10.1016/j.neuroimage.2021.118086

**Published:** 2021-08-01

**Authors:** Martyn Pickersgill

**Affiliations:** University of Edinburgh, Usher Institute of Population Health Sciences and Informatics, Medical School, Teviot Place, Edinburgh EH8 9AG, United Kingdom

**Keywords:** Neuroimaging, Neuroscience, Social dimensions and implications, LMICs

## Abstract

Drawing on insights from sociology, anthropology, and the history of science and medicine, this paper considers some of the social dimensions and implications for neuroimaging research undertaken within low- and middle-income countries (LMICs). It highlights three key inter-connected issues: (1) technologies for enhancing understandings of ill-health are theory-laden; (2) such technologies are theory-generating; and (3) studies of mental ill-health can also introduce new idioms for understanding subjective distress. The paper unpacks and explores these issues. It argues that the use of neuroimaging technologies in population research has the potential to contribute to solidifying – or even introducing – a biological (and specifically brain-based) understanding of mental ill-health within the communities under study. Examples from studies of neuroscience and society in various high-income countries (HICs) where neuroimaging is popular within public discourse illustrates how this can happen, and with what effects. The social dimensions and implications of neuroimaging are issues that all researchers using these technologies need to not only anticipate, but also explicitly plan for (and potentially seek to mitigate). Without adequate consideration, neuroimaging research carries with it particular risks in relation to extending the epistemological coloniality associated with HIC-sponsored studies conducted within LMIC settings.

## Highlights

• Considers the social dimensions/implications for neuroimaging research in LMICs.
• Technologies for enhancing understandings of ill-health are theory-laden.
• Such technologies are theory-generating.
• Studies of mental ill-health can introduce new idioms for understanding subjective distress.
• Researchers must anticipate and potentially mitigate the social implications of neuroimaging.

## 1 Introduction

Within many high-income countries (HICs), research sponsors are actively seeking to cultivate not only technoscientific innovation within low- and middle-income counties (LMICs), but also the catalysis of social change. Such research can risk the reproduction of colonialism (Noxolo, 2017), and in particular epistemological colonialism (Hlabangane, 2017; Ndlovu-Gatsheni, 2012). This paper explores some of the social dimensions and implications for neuroimaging research driven or supported by HICs and undertaken within LMICs. In particular, it draws on existing sociological, anthropological, and historical research to highlight three key connected issues. First, technologies for enhancing understandings of ill-health are theory-laden. Second, such technologies are theory-generating. Third, studies of mental ill-health can also introduce new idioms for understanding subjective distress. Consideration of these issues (unpacked below) is vital when undertaking studies in settings wherein particular (psycho)pathological categories of research interest and the use of specific neuroimaging technologies to research them are marginal, nascent, or otherwise unfamiliar.

## 2 Exploring the social dimensions and implications of neuroimaging research

Research generates not only new knowledge, but also new interactions between individuals and institutions - and potentially stimulates new legal and policy frameworks. Science is thus an intrinsically social enterprise, including in relation to how research agendas are set, teams built, and credit apportioned (Latour and Woolgar, 1986). Robust scholarship in sociology, anthropology, and history has extensively investigated the social dimensions of science (Jasanoff et al, 1995). This is drawn upon below to explicate and illustrate some of the key social dimensions and implications of neuroimaging research.

### 2.1 Technology as theory-laden

Technologies are sponsored, developed, marketed, and operated by people within and across societies: technology, like science, is thus a social enterprise. Accordingly, it can carry with it the values and assumptions of the contexts in which it is imagined, incorporated, and implemented (Winner, 1980; Benjamin, 2019). This leads to the first key point advanced in this paper: technologies for enhancing understandings of ill-health are theory-laden. By this, it is meant that biomedical technologies carry with them particular assumptions about the nature of the body and of pathology (Lock and Nguyen, 2011). These define the operations and potential utility of technologies.

In what senses are neuroimaging technologies like PET and fMRI theory-laden? For a start, they assume the relevance of the brain to the self and its pathologies (Dumit, 2004), with neuroimages becoming “sites for excavating biological reality/pathology” (Prasad, 2005: 292). As historians have demonstrated, the siting of subjectivity within the brain is a product of particular social and technological histories (Vidal, 2009). In the case of neuroscientific research into experiences regarded in many HICs as psychiatric disorder, the use of technologies like fMRI almost inevitably come to further inflame debate within psychiatry and beyond around the place, role, and impact of (neuro)biology in aetiology (Bertorelli, 2016). In contexts where the brain is not, a priori, taken to be of singular import to constructions of selfhood, gaps can feasibly emerge between the perspectives of neuroscientists and those of collaborating clinicians and research participants. The questions such disjuncture pose include the extent to which participant autonomy could be compromised by this kind of mismatch between perceptions of selfhood, the brain, and the power and potential of neuroimaging (Pickersgill, 2011).

### 2.2 Technology as theory-generating

As well as being theory-laden, technologies are theory-generating. In other words, the use of technologies of visualization help to embed ideas about the body and pathology within not only the research studies in which they are employed, but also within the understandings and perspectives of those who come into contact them. This might be directly - e.g., as study participants - or indirectly, such as through the media or through healthcare. Buchbinder (2015) provides a compelling example of how neuroimaging research is reimagined within US pain clinics when professionals communicate with adolescent patients. This includes through hopeful metaphors to young people that, for instance, “held the promise of possible futures in which new neural connections could erase the work of intractable pain” (Buchbinder, 2015: 310). In Australia, Barnett et al. (2018, 2020) have demonstrated how the use of neuroscientific technologies within research into drug addiction has contributed to a discourse of a ‘hijacked brain’ within clinical addiction practice, which has helped to generate new moral positions for people living with addiction. In societies where individuals are often imagined to be self-contained units governed predominantly through ‘personal responsibility’, allusions to a ‘hijacked brain’ can absolve people for actions regarded as inappropriate or illegal. This can have beneficial effects, while also orientating recovery towards the recovering brain per se (as opposed to more psychosocial formulations).

Cohn's (2010) UK-sited research with patient-participants in mental health research using neuroimaging demonstrates the theory-generating (or at least theory-reifying) potential of technologies like fMRI. In interviews following a scan where participants were granted a souvenir of a neuroimage, people commonly treated this as a straightforward picture of their brain that could precisely localize their condition, “allowing [the brain] to be conceived as an alien pathological entity” (Cohn, 2010: 75; see relatedly Rapp, 2011). In their work in Canada with people diagnosed with mood disorders, Buchman and colleagues (2013) demonstrate how neuroimaging research can be read as both potentially destigmatizing and legitimizing of experience *and* as likely to reify particular diagnoses and to more tightly couple the relationship between pathology and identity. The findings of Buchman et al. (2013) exemplify the ambivalent nature of the social implications of neuroimaging (see also Whiteley et al, 2017). More specifically: the theories that are generated through technology use and which circulate within professional, patient, and popular discourses have neither straightforwardly positive nor negative effects. Consequently, they require careful and context-specific disentangling and engagement.

### 2.3 New idioms of distress

Alongside being theory-laden and theory-generating, the use of neuroimaging technologies in studies of mental ill-health can also introduce new idioms for understanding subjective distress. This potential relates to the purposes of the study for which neuroimaging is being used, and the other tools being employed as part of the research infrastructure. For example, diagnostic categorisations from the US Diagnostic and Statistical Manual of Mental Disorders (DSM) can be introduced through research practices. Yet, this text is “embedded in a very specific [US] biomedical epistemology” (Behrouzan, 2016: 27), and its introduction sometimes generates ambivalence and friction within clinical settings where “global psychiatry and local psychiatries” (Kitanaka, 2012: 81) are made to share space. New idioms for understanding subjective distress are themselves theory-laden: they contain remnants of the social and epistemic contexts of their generation. Like technologies, particular assumptions come with them about the nature of body and mind, and the ways these (inter)act to produce experiences of distress. Further, new idioms of distress can be theory-generating: they can be picked up by a range of people (i.e., not solely scientists and clinicians) and informally applied to others. Notions of distress crafted largely, though not exclusively, in North America can become accommodated elsewhere in the world in processes of what Duncan (2018) terms ‘psy-globalization’ (see relatedly Cox and Webb, 2015; Davar, 2014; Mills, 2013). In the UK, a key example of the adoption of US diagnostic terminology is talk of attention deficit hyperactivity disorder (ADHD) in educational contexts. Teachers sometimes informally describe students they consider to be particularly restless and impulsive as “a bit ADHD”; the pupils then come to be treated differently by teachers, with potentially detrimental effects (Armstrong et al, 2016: 15). The neurobiological understandings that underpin such informal characterisations are themselves propelled through the use of neuroimaging technologies and the widespread cultural circulations of texts and iconography associated with these.

There is ample evidence of how DSM diagnostic categories like depression have become entwined with pre-existing categories (such as *shenjing shuairuo* in China; Lee, 1999) and psychopharmacuetical treatments interweaved with existing ontologies of (ill-)health (e.g., the reference of SSRIs as ‘mind food’ in parts of India; Ecks, 2014). As Kohrt et al. (2016) observe, research is needed into how psychopharmaceuticals come to be assimilated into people's lives following their introduction within contexts where they were previously less readily available. Particular attention must be paid to (a) how new patterns of consumption in LMICs relate to the introduction of categories of distress from HICs (e.g., the US), and (b) how neuroimaging technologies and the research and discourse they enable are purposively leveraged or implicitly presented as a means to make sense of these categories. This is especially important given concerns that attempts to innovate mental healthcare within LMICs can or might contribute to problematic forms of (bio)medicalization (Davar, 2014; Mills, 2018; Ventevogel, 2014; White and Sashidharan, 2014). The extent to which neuroimaging research can act as a vehicle for introducing new idioms of distress, and contribute to forms of biomedical imperialism and colonialism (cf. Ugwu, 2019), should be subject to close consideration. However, such considerations cannot themselves become a vehicle for a form of epistemological coloniality within which it is simply assumed that concepts and technological from HICs will necessarily have transformative effects in LMICs.

## 3 Conclusion

Alongside the ethical and legal aspects and implications of neuroimaging technologies (Palk et al., 2020), the social dimensions and ramifications of research employing these tools also demands attention - whether studies make use of either long established or newer techniques like optical tomography (Fishell et al., 2020). Through considerations of the existing social scientific evidence base, this paper has argued that the use of neuroimaging technologies can contribute to solidifying – or even introducing – a biological (and specifically brain-based) idiom for, and set of understandings of, mental ill-health and subjective distress.

As existing scholarship has demonstrated, such introductions and forms of reification have happened in a range of nations. At the same time, this research has also revealed that there are limits to the extent to which accounts of the self and of distress have been neurobiologized as a consequence of the proliferation of neuroimaging (Barnett et al., 2020; Broer et al., 2020; Pickersgill et al., 2011). People - including, of course, neuroscientists themselves - are often subtle and creative in whether and how they ascribe significance to the brain and imaging technologies. However, caution is nevertheless warranted about disjunctures in understandings between scientists and study participants which could compromise the autonomy of the latter. Scientists also need to be mindful of how and in what ways so-called ‘local’ knowledge about the self is encouraged via imaging research to reform around purportedly ‘global’ neurobiological understandings. The salience of this issues relates to the wider potential for HIC-funded research in LMICs reproduce and extend forms of biomedical and epistemological colonialism (Kalinga, 2019; Ndlovu-Gatsheni, 2012).

This commentary resists offering straightforward guidelines for how researchers might deal with the proliferation of categories and ontologies beyond neuroimaging research sites. This is not least as a consequence of the heterogeneity of LMICs (and HICs), and hence the redundancy of advancing universal recommendations that are unlikely to always resonate sufficiently with the particularities of national contexts. Nevertheless, it is argued that these matters are part of the range of social and ethical issues that researchers need to not only anticipate but also explicitly plan for (and potentially seek to mitigate). Such consideration and planning will benefit from close collaboration with social scientists (Pickersgill et al., 2018) – as well, of course, with the communities with which neuroscientists should be co-producing their research questions and study designs (Nyirenda et al., 2020).

Scientists undertaking neuroimaging research need to be attentive to the social dimensions and implications of their work. This is particularly the case when undertaking studies that: (a) employ imaging technologies in populations for whom the research techniques and the pathological categories of research interest are marginal, nascent, or otherwise unfamiliar (especially for groups where stigmatization is a possible outcome of the movement of scientific findings into wider society; e.g. paediatric populations (Wedderburn et al., 2020; Xie et al., 2020) and older adults (Bachli et al., 2020; Farina et al., 2020)); and (b) have the potential to induce uncertainties and even alarm among participants and their families (such as imaging research into infant neurodevelopment; Katus et al., 2020; Turesky et al., 2020).

## NeuroImage – author credit statement

I am the sole-author of the manuscript, which I conceived and wrote in full.

## Data and code availability statement

The article does not draw on primary neuroscientific data nor use code.

## Declaration of Competing Interest

None
